# A Mixed Comparisons of Different Intensities and Types of Physical Exercise in Patients With Diseases Related to Oxidative Stress: A Systematic Review and Network Meta-Analysis

**DOI:** 10.3389/fphys.2021.700055

**Published:** 2021-08-05

**Authors:** Zhenghui Lu, Yining Xu, Yang Song, István Bíró, Yaodong Gu

**Affiliations:** ^1^Faculty of Sports Science, Ningbo University, Ningbo, China; ^2^Doctoral School on Safety and Security Sciences, Obuda University, Budapest, Hungary; ^3^Faculty of Engineering, University of Szeged, Szeged, Hungary

**Keywords:** physical exercise, antioxidants, oxidative stress, chronic diseases, network meta-analysis

## Abstract

The balance of oxidative and antioxidant systems is of great importance to the human body. Physical exercise, as one of the ways to improve physical health, seems to modulate this balance. However, different intensities and types of physical exercise have other effects on the treatment of unhealthy people. To understand the impact of exercise training on the oxidative and antioxidant systems of adults with oxidative stress-related disorders, a network meta-analysis was used to compare the mixed effects of different intensities and types of exercise training. This systematic review included all eligible RCTs from PubMed, Medline, Cochrane Library, and CINAHL. Eleven of the studies met the inclusion criteria (at study completion, *n* = 666 participants). Seven studies reported that the level of MDA decreased significantly after exercise (*p* < 0.05), and 3 studies reported that the level of SOD increased significantly after exercise (*p* < 0.05). In conclusion, long-term high-intensity aerobic training and Tai Chi or Yoga can effectively improve oxidative stress in unhealthy people. In addition, different types of diseases on the effect of exercise intervention seems to be other, diabetes and chronic kidney patients using moderate-intensity aerobic training or Tai chi and Yoga effect are better; Moderate-intensity aerobic training had a better impact on OS improvement in patients with irritable bowel syndrome and severe depression. However, more research is needed to determine the effects of different levels and types of physical activity on oxidative stress in unhealthy populations.

**Systematic Review Registration:** PROSPERO identifier: CRD42021242025. https://www.crd.york.ac.uk/prospero/.

## Introduction

Oxidative stress (OS) would occur when the oxygen metabolism by-products were produced and accumulated and eventually beyond oxidation-resist ability (Sies, 1991, 1997; Newsholme et al., 2016; Pizzino et al., 2017; Powers et al., 2020). There is evidence that OS has a close relationship with the pathogenesis of various diseases (Taniyama and Griendling, 2003). For example, OS is an essential factor in myocardial infarction (Yan et al., 2018), which may also lead to liver damage (Rigamonti et al., 2003) and be a potential risk factor of diabetes and cardiovascular disease (Locatelli et al., 2003; Newsholme et al., 2016), and also related to the pathogenesis of hypertension (Briones and Touyz, 2010). Therefore, the biomarkers of OS need to be paid more attention to in treating these diseases. The primary mechanism for OS production is that the defect of mitochondria, which leads to the peroxidation damage of mitochondrial membrane, decreases cytochrome C oxidase activity (Chen and Yu, 1994; Navarro et al., 2001, 2004; Konopka and Nair, 2013), resulting in overactive species. When there is excessive production of active species, especially reactive oxygen species (ROS) and active nitrogen species (RNS), it will cause severe damage (Newsholme et al., 2016), for example, the destruction of cell structure, lipids, proteins and genetic material (Islam, 2017).

Although the current research suggests that OS is harmful to the human body, a certain ROS and RNS level is significant for maintaining the regular operation of the body (Valko et al., 2007; Pizzino et al., 2017). For example, protein phosphorylation processes and the activation of some transcription factors must be carried out in a certain level of ROS environment (Rajendran et al., 2014), ROS and RNS also play an important role in preventing infection, etc. (Valko et al., 2007). Therefore, how to maintain a specific operating system has important research value. Therefore, the research on how to maintain a specific group of OS is precious.

Physical exercise is the common intervention studied to turn OS in the body for the better. Physical exercise improves heart and lung health and mental health and is a powerful tool for preventing and treating various chronic diseases (Ruegsegger and Booth, 2018). And people with exercise habits usually have a lower all-cause mortality rate (Gulati et al., 2003; Mora et al., 2003; Myers et al., 2004; Kokkinos et al., 2008). However, physical exercise can promote health, high-intensity training may lead to OS (Magherini et al., 2019). In the 1970s, Dillard et al. (1978) showed that a 60 min cycling exercise under the intensity of 50% *VO*_2max_ could lead to an increase in lipid peroxides, and subsequent studies also found that physical exercise changes the levels of some oxidizing biomarkers (Ramos et al., 2015; Gomes et al., 2017). Subsequently, Davies et al. (1982) discovered that skeletal muscle contraction produces oxidation markers ROS. At present, the source of physical exercise increasing ROSor RNS is not completely clear (Thirupathi et al., 2018). Still, we already know that strenuous repetitive exercise causes muscle damage (Barbe and Barr, 2006), which destroys cells and leads to intracellular biochemical changes, resulting in ROS (Powers and Jackson, 2008).

Although exercise leads to an increase in oxidation indicators in the human body, the body's antioxidant defense system is also up-regulated to resist the beneficial adaptation of the body guided by OS (Polidori et al., 2000; Fisher-Wellman and Bloomer, 2009). Many studies have shown that OS levels and antioxidant capacity change after acute exercise (El Abed et al., 2011; Arazi et al., 2021; Bouviere et al., 2021), and there is a lot of evidence that long-term exercise habits could weaken OS (De Sousa et al., 2017). On the other hand, increasing the body's antioxidants activity may increase antioxidant capacity (Halliwell and Whiteman, 2004). As mentioned above, OS may cause cell damage and is closely linked to various diseases' pathogenesis. From the long-term perspective, maintaining a certain intensity of physical exercise habits may reduce the risk of stroke, cardiovascular disease, and coronary heart disease by improving oxidative balance and improving sick people's health.

In addition, different intensity and types of exercise seem to have different effects on OS, especially in people with chronic diseases. It has been proved that more than a certain intensity of single exercise increases ROS production and leads to injury (Radák et al., 1999; Gomes et al., 2017). As far as the current situation is concerned, people have already had a certain understanding of exercise-induced OS. However, most studies were conducted in healthy people, there is still a lot of controversy about the impact of an exercise intervention on unhealthy people (Larsen and Matchkov, 2016; Poblete-Aro et al., 2018; Farzanegi et al., 2019; Silva et al., 2019). The evidence obtained is still contradictory, and the heterogeneity may come from different experimental designs. Some practical schemes also include the intake of antioxidant drugs, which is relatively difficult to compare. At present, there is no industry-recognized best scheme for OS for unhealthy people.

Therefore, we hypothesized that different intensities and types of physical activity may have other effects on OS in unhealthy people and that there may be an optimal intervention plan. Therefore, in this review, we will study the impact of different intensities and types of physical exercise on OS in unhealthy people.

Detection of OS biomarkers is a common method to assess human OS status (Marrocco et al., 2017). At present, there are various measurement methods, and the selection of indicators in the experiment usually depends on the purpose and design of the research (Marrocco et al., 2017). In this review, Malondialdehyde (MDA) and superoxide dismutase (SOD) were selected as the primary outcome measures of OS and antioxidant capacity.

To better understand the effect of exercise on the unhealthy population, this review aims to make indirect and mixed comparisons of the interventions on OS marker (MDA) and antioxidant marker (SOD) in unhealthy people using a network meta-analysis method. So far, no studies have adjusted and mixed comparisons. We implemented adjusted and mixed verbs with network meta-analysis, attempting to provide better advice for the unhealthy population from OS.

## Methods

This review was conducted based on the Preferred Reporting Items for Systematic Reviews and Meta-Analysis guidelines (PRISMA). Literature collection, exclusion criteria, and retrieval strategies are jointly proposed and agreed upon by two authors (Zhenghui Lu and Yining Xu) and established a priori to minimize bias.

### Registration

Our research program has been registered on Prospero, the International Register of Expectations for System Evaluation; Registration number: CRD42021242025.

### Eligibility Criteria

Only studies of randomized controlled trials published from 2000 to 2020 would be included in this review. In addition, the research must be published in English and reviewed by other peers. The population, interventions, comparisons, and outcomes of this review were as follows.

#### Participants/Population

All subjects included in this study were adults with diseases related to OS.

#### Intervention(s)

This review's long-term physical exercise interventions included aerobic training, aerobic mixed resistance training, Tai Chi, Yoga, etc. This review excludes studies with only one exercise intervention and defines other studies as long-term studies.

According to the classification of previous studies, In this review, the criteria for the variety of High-intensity training are (1) exercise with an average heart rate ≥ 80% maximum heart rate (max HR); (2) exercise with a metabolic equivalent (METs) > 7. Moderate intensity training classification criteria are (1) exercise with an average heart rate between 55 and 80% max HR; (2) aerobic bicycles with an average power of 55–60% peak power. Low-intensity training classification criteria are (1) exercise with an average heart rate of <55%; (2) Tai Chi and Yoga usually have a maximum oxygen uptake of around 40% *VO*_2_ (Medicine ACoS, 2013), so they are also classified as low intensity training.

In addition, of the 5 studies that used heart rate as an index of intensity, the maximum heart rate in 3 studies was estimated by age or resting heart rate (Gordon et al., 2008; Wycherley et al., 2008; Schuch et al., 2014), and the other 2 did not elaborate on (Chen et al., 2010; Boff et al., 2019), so more accurate information could not be obtained.

#### Comparator(s)/Control

The above interventions' indirect comparisons are feasible because the network meta-analysis is based upon Bayes' theorem (Mills et al., 2013). The comparator(s)/control criteria were the same as the intervention(s) criteria.

#### Outcomes

This review's outcome indicators are MDA, a biomarker commonly used to judge the OS level, and a biomarker of antioxidant capacity SOD.

Other indicators included in this review were too rare or had different detection methods to perform a reticular meta-analysis, so they were treated as secondary indicators for supplementary analysis. These indicators included: pro-oxidation indexes phospholipase A2 (PLA2), protein oxidation (POX), ROS, NO, and PC; the antioxidant indexes were SOD, Catalase, thyroid-stimulating hormone (TSH), free thyroxine (FT4), glutathione peroxidase (GPX), 8-isoprostane (8-ISO), F2-isoprostanes (F2-ISO) and Trolox equivalents.

In addition, among the 11 studies included in the review, the blood collection sites of 8 studies were collected from the anterior cubital vein (Gordon et al., 2008, 2013; Wycherley et al., 2008; Chen et al., 2010; Schuch et al., 2014; Mallard et al., 2017); in one study, blood was collected from the lateral vastus muscle (Linke et al., 2005); in one study, blood was collected during hemodialysis (Wilund et al., 2010), and the collection site of blood was not clearly identified in 3 studies (Luk et al., 2012; Maleki et al., 2018; Boff et al., 2019).

### Information Sources

This review uses PubMed, Medline, Cochrane Library, and CINAHL to conduct a comprehensive and repeatable literature search before December 2020. If the data is insufficient, the author will be contacted to ask to provide the exact data.

### Search Strategy

1. In PubMed, the search term was “((oxidative stress [Title/Abstract]) OR (oxidant stress [Title/Abstract])) NOT ((protocol [Title]) OR (design [Title])) AND ((randomized [Title/Abstract]) [OR (randomized [Title/Abstract]))”;
2. In Medline, Cochrane Library, and CINAHL, the search term was “(oxidative stress OR oxidant stress TI) OR (oxidative stress OR oxidant stress AB) NOT (protocol OR design TI) AND (randomized OR randomized AB).”

The title's selection, abstract and full text is jointly completed by two independent authors (Zhenghui Lu and Yining Xu). The differences will be judged by a third independent arbitrator (Yang Song).

### Study Selection

The process of screening the abstract and the text is done by two independent authors (Zhenghui Lu and Yining Xu). When no opinion can be reached, the disagreement will be judged by a third independent arbitrator (Yang Song).

Studies would be excluded if they meet the following conditions: (1) Studies with healthy subjects or minors; (2) Studies which only performed one-time exercise; (3) Studies using invasive interventions such as surgery and injections; (4) Studies in which specific data of outcome indicators are not provided, or where the authors do not receive timely answers.

### Data Collection Process

All potential studies were downloaded and imported into Endnote X9 (Thomson Reuters, Carlsbad, California, USA), and duplicated tasks were deleted. The data collection is done by two independent authors (Zhenghui Lu and Yining Xu). When the opinions cannot be reached, the third independent arbitrator will judge the differences (Yang Song). The information included demographic characteristics (average age and gender), clinical characteristics (Body mass index), details of experimental design (sample size, intervention method, and follow-up time), and outcome indicators.

### Data Items

The study's funders did not contribute to design or implementation. Therefore, the author is fully responsible for data collection, analysis, interpretation, and reporting. Corresponding authors have access to all data and are ultimately responsible for the submission of publications.

### Risk of Bias in Assessment

The risk of bias is evaluated by two evaluators (Zhenghui Lu and Yining Xu) using the Cochrane Collaboration Risk of Bias Assessment Tool. When no agreement can be reached, the disagreement will be judged by a third independent arbitrator (Yang Song).

### Summary Measures

The data preprocessing and analysis were made by two independent investigators (Zhenghui Lu and Yining Xu). Microsoft Excel (Version 16.0, Microsoft Corporation, Redmond, WA, USA) was used to preprocess the original data and convert the results into average and standard deviation (Mean ± SD).

The processed data are analyzed by Aggregate Data Drug Information System (ADDIS V1.16.8 produced by Drugis.org, http://drugis.org/software/addis/index), calculated the effect size, the data are aggregated into the network meta-analysis, and all the graphs and results were output. The results of the network meta-analysis are introduced in the following parts.

## Result

### Search Strategy and Information Extraction

**Figure 1:**
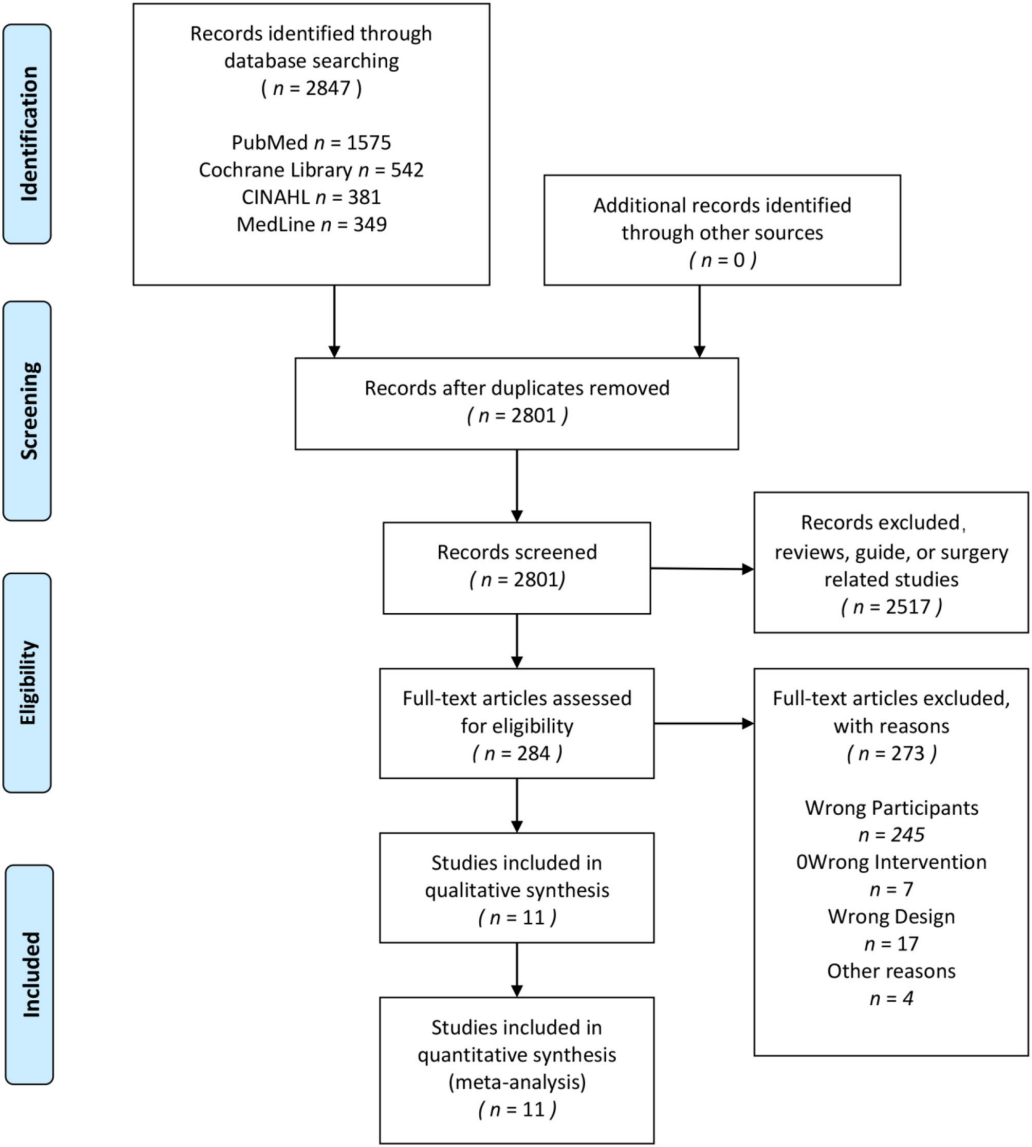
PRISMA flow diagram for the systematic review and network meta-analysis.

A total of 2,754 studies were searched for screening through the electronic search of four scientific databases. After filtering by title and abstract, 2,470 articles were excluded. Eleven studies (All subjects were between the ages of 18 and 77; diseases included in the review had type 1 diabetes, type 2 diabetes, chronic heart failure, coronary heart disease, irritable bowel syndrome, major depression, and chronic kidney disease) were included in the final analysis. The details of the article filtering process would be shown in Figure 1. The information of all included studies would be shown in Table 1.

**Table 1:** The study characteristics of included studies.

**References**	**Participants**	**Mean age** ** (Range)**	**Gender** ** (Female/All)**	**Total duration; wkly frequency**	**Intervention**	**Blood collection site; sampling time**	**Pro-oxidant**	**Antioxidant**
Boff et al. (2019)	Patients with type 1 diabetes	23.5 (18–34)	15/27	8 weeks; 3/week	HIAT	Unknow	MDA (↔)	
				8 weeks; 3/week	MIAT		MDA (↔)	
					BLANK		MDA (↔)	
Chen et al. (2010)	Patients with type 2 diabetes	58.3 (40–70)	59/94	12 weeks; 3/week	Tai Chi	Anterior cubital vein; the time for blood collection was 7: 30 am-9: 00 am, and no intervention was carried out on the same day.	MDA (↓); PLA2 (↔); POX (↔)	
				12 weeks; 3/week	LIAT		MDA (↔); PLA2 (↔); POX (↔)	
Gordon et al. (2013)	Patients with chronic kidney disease	41.7 (20–70)	Unknow/68	16 weeks; unknow	Yoga	Anterior cubital vein; the time for blood collection was 7: 30 am-9: 00 am, and no intervention was carried out on the same day.	MDA (↓); PLA2 (↓); POX (↓)	SOD (↑); Catalase (↑); TSH (↓); FT4 (↑)
					BLANK		MDA (↓); PLA2 (↓); POX (↑)	SOD (↓); Catalase (↓); TSH (↓); FT4 (↑)
Gordon et al. (2008)	Patients with type 2 diabetes	63.8 (40–70)	186/231	12 weeks; 3–4/week	MIAT	Anterior cubital vein; the time for blood collection was 7: 30 am-9: 00 am, and no intervention was carried out on the same day.	MDA (↔); PLA2 (↔); POX (↔)	SOD (↔); Catalase (↔)
				24 weeks; 3–4/week	MIAT		MDA (↓); PLA2 (↔); POX (↔)	SOD (↑); Catalase (↔)
				12 weeks; 3–4/week	Yoga		MDA (↔); PLA2 (↔); POX (↔)	SOD (↔); Catalase (↔)
				24 weeks; 3–4/week	Yoga		MDA (↓); PLA2 (↔); POX (↔)	SOD (↑); Catalase (↔)
					Blank		MDA (↔); PLA2 (↔); POX (↔)	SOD (↔); Catalase (↔)
Linke et al. (2005)	Patients with chronic heart failure	53.6 (49–60)	0/23	24 weeks; 4–6/week	MIAT	Lateral vastus muscle; unknow		SOD (↔); GPX (↔); Catalase (↔)
					Blank			SOD (↔); GPX (↔); Catalase (↔)
Luk et al. (2012)	Patients with stable coronary artery disease	67.2 (59–77)	16/64	8 weeks; 3/week	HIART	Unknow		SOD (↔); F2α (↔)
					Blank			SOD (↔); F2α (↔)
Maleki et al. (2018)	Patients with irritable bowel syndrome	34.0 (18–41)	51/51	12 weeks; 4–6/week	MIAT	Unknow; stop exercise at least 24 h before blood collection	MDA (↔); ROS (↓); NO (↓)	SOD (↑); GPX (↑)
				24 weeks; 4–6/week	MIAT		MDA (↓); ROS (↓); NO (↓)	SOD (↑); GPX (↑)
					Blank		MDA (↔); ROS (↔); NO (↔)	SOD (↔); GPX (↔)
Mallard et al. (2017)	Patients with type 2 diabetes	57.0 (44–65)	14/36	52 weeks; 3/week	HIAT	Anterior cubital vein; no strenuous exercise for at least 24 h before blood collection	PC (↔)	TEAC (↔); GPX (↔); F2α (↔)
				52 weeks; 3/week	MIAT		PC (↔)	TEAC (↔); GPX (↔); F2α (↔)
Schuch et al. (2014)	Patients with severely depressed inpatients	42.7 (18–60)	19/26	3 weeks; 3/week	MIAT Blank	Anterior cubital vein; blood collection time is 10: 30–11: 30 in the morning. It is unknown whether exercise is carried out before blood collection.	MDA (↓) MDA (↑)	
Wilund et al. (2010)	Patients with chronic kidney disease	44.0 (30–70)	9/17	16 weeks; 3/week	MIAT Blank	Blood was collected from patients' dialysis lines during regularly scheduled (monthly) blood collection times at the clinic; the time for blood collection is 48–72 h after exercise.	MDA (↓) MDA (↔)	
Wycherley et al. (2008)	Patients with type 2 diabetes	52.4 (33–62)	29/29	12 weeks; 4–5/week	MIAT	Anterior cubital vein; unknow	MDA (↓)	Trolox equivalents (↔)
					Blank		MDA (↓)	Trolox equivalents (↔)

*Blank, Blank control; HIAT, High intensity aerobic training; MIAT, Medium intensity aerobic training; LIAT, Low intensity aerobic training; HIART, High intensity aerobic mixed resistance training; MDA, Malondialdehyde; SOD, Superoxide dismutase; PLA2, Pospholipase A2; POX, Protein oxidation; ROS, Reactive oxygen species; NO, Nitric oxide; PC, Protein carbonyl; TSH, Thyroid-stimulating hormone; FT4, Free thyroxine; GPX, Glutathione peroxidase; 8-iso, 8-isoprostane; F2-iso, F2-isoprostanes*.

### Risk of Bias

**Figure 2:**
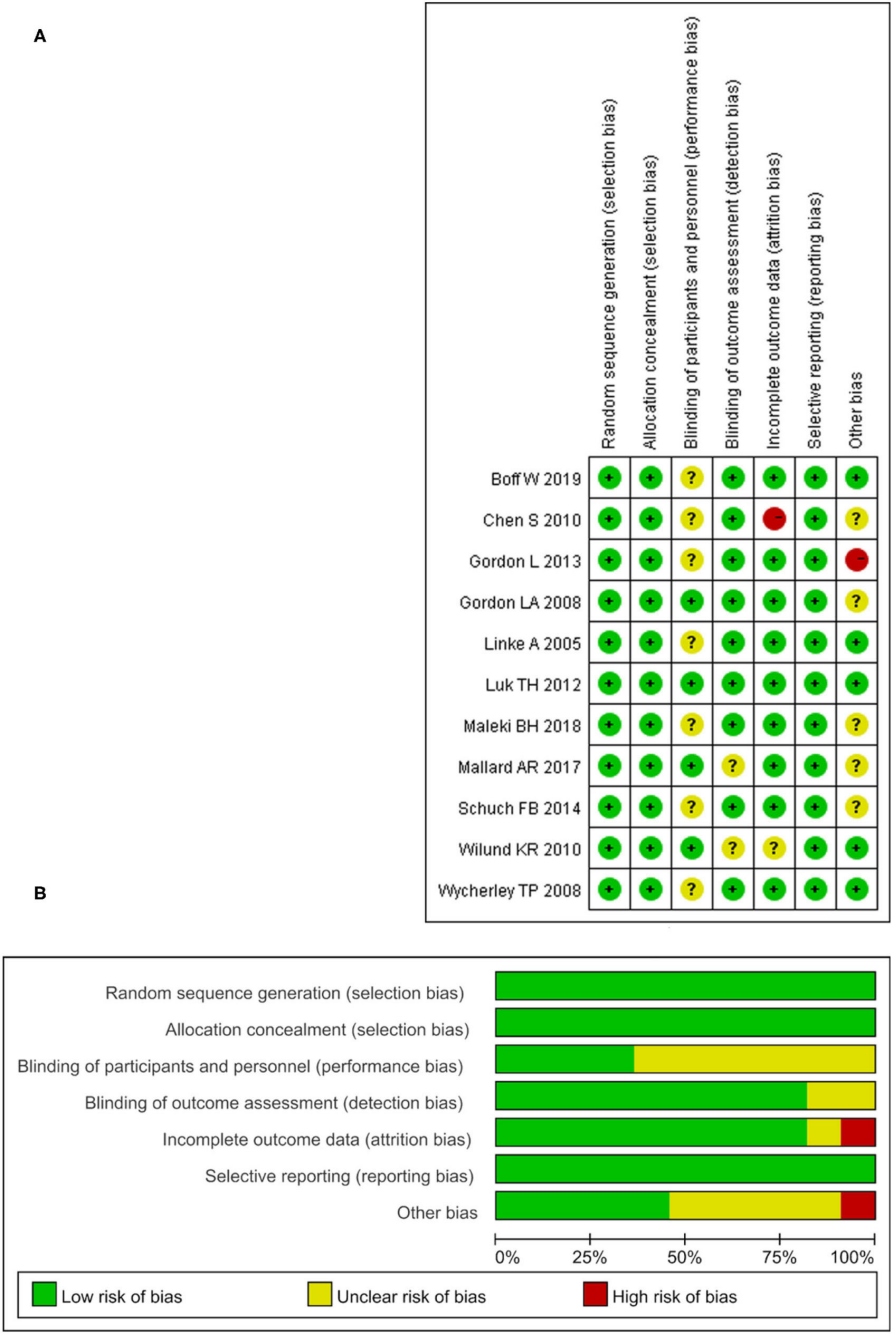
The result of the risk of bias assessment. **(A)** Risk of bias graph; **(B)** Risk of bis summary.

The risk of bias in the 11 included studies was assessed, and the consensus was reached after discussion. The overall result is shown in Figure 2. Participants' randomization and concealment methods were well-reported in all studies. Sixty-four percentage of studies did not adequately describe participant or staff blinding. Eighteen percentage of the studies did not describe whether the evaluator was blind. Only one study had incomplete results due to subjects dropping out (Chen et al., 2010), and the other did not fully account for them (Wilund et al., 2010). All the studies recorded their research plan and researched according to the program.

### Network Meta-Analysis

#### Pro-oxidant Marker

**Figure 3:**
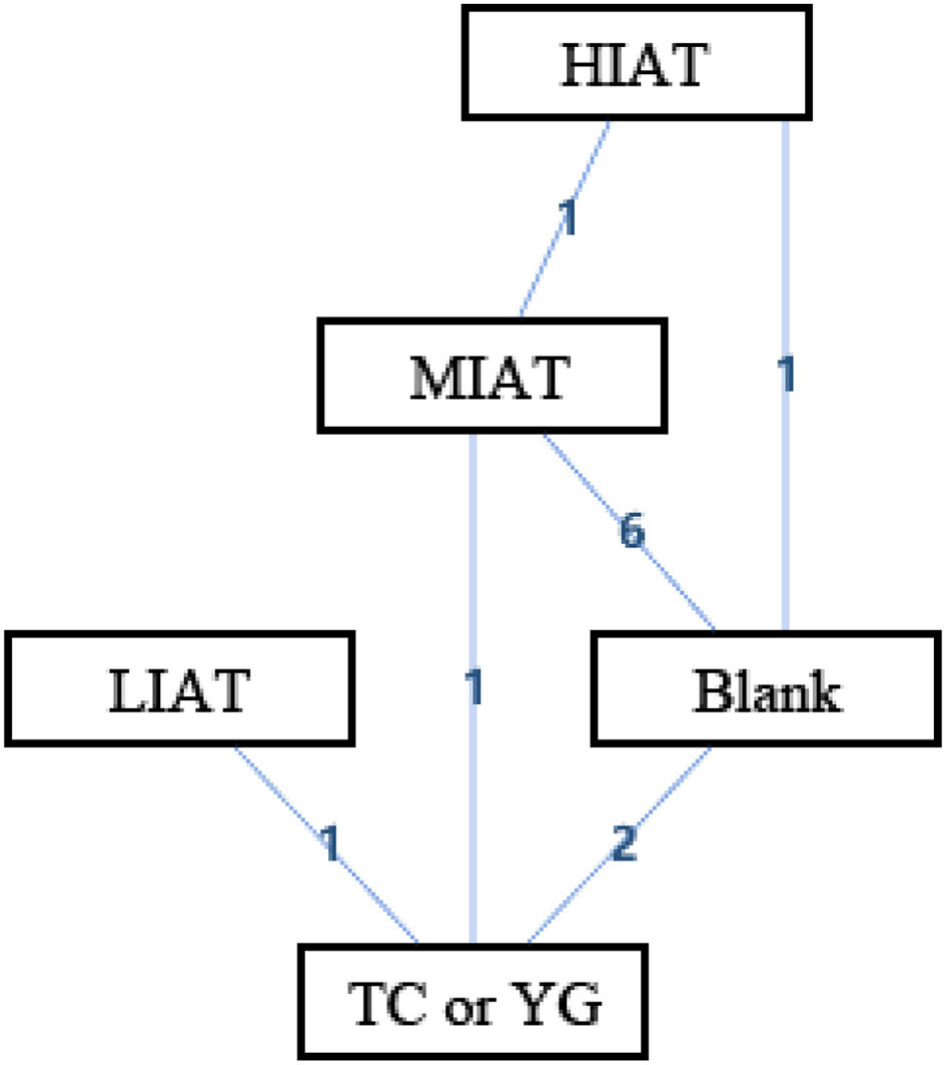
The network structure of intervention of malondialdehyde (MDA). Blank, Blank control; HIAT, High intensity aerobic training; MIAT, Medium intensity aerobic training; LIAT, Low intensity aerobic training; TC or YG, Tai Chi or Yoga. The numbers on lines represent the number of studies that make direct comparisons between interventions.

Figure 3 shows the geometry of the acute intervention network of MDA, a biomarker of OS in unhealthy populations. As shown in the figure, there is a mixed intervention comparison (Figure 3). The mixed intervention comparison developed from the traditional Meta analysis, and expanded from the standard double-arm test Meta analysis to a series of different treatment factors to analyze and compare each other and synthesize at the same time. The mixed intervention comparison includes direct comparison and indirect comparison. Because the evidence is a closed-loop, the inconsistency of the evidence should be evaluated.

In the mixed comparison of high-intensity aerobic training (HIAT), moderate-intensity aerobic training (MIAT), low-intensity aerobic training (LIAT), Blank and Tai Chi or Yoga, the random effect standard deviation of the consistency model and its 95% confidence interval was 1.23 (0.18, 5.94), the random effect standard deviation of the inconsistency model and its 95% confidence interval was 2.43 (0.21, 7.09). The inconsistency model's standard inconsistency deviation and 95% confidence interval were 2.55 (0.10, 9.13). In addition, the inconsistency factors of MIAT, Blank, and Tai Chi or Yoga were 0.03 (−7.03, 7.39). There were significant differences in the standard deviations of random effects between the congruent and incongruent models. The inconsistency factor in the intervention cycle is close to 0. It means that there may be consistency differences related to specific nodes, and node split analysis is required.

Table 2 is a sorted table of the network geometry of Figure 3.

**Table 2:** The League Table of the interventions for the malondialdehyde (MDA).

HIAT				
−0.37 (−8.24, 7.18)	LIAT			
0.05 (−4.40, 5.35)	0.33 (−5.26, 7.60)	MIAT		
−0.54 (−5.76, 3.75)	−0.13 (−6.69, 5.87)	−0.53 (−3.65, 0.77)	Blank	
−0.20 (−6.21, 5.30)	0.17 (−4.94, 5.43)	−0.17 (−4.61, 2.82)	0.30 (−2.90, 4.05)	TC or YG

*Blank, Blank control; HIAT, High intensity aerobic training; MIAT, Medium intensity aerobic training; LIAT, Low intensity aerobic training; TC or YG, Tai Chi or Yoga*.

**Figure 4:**
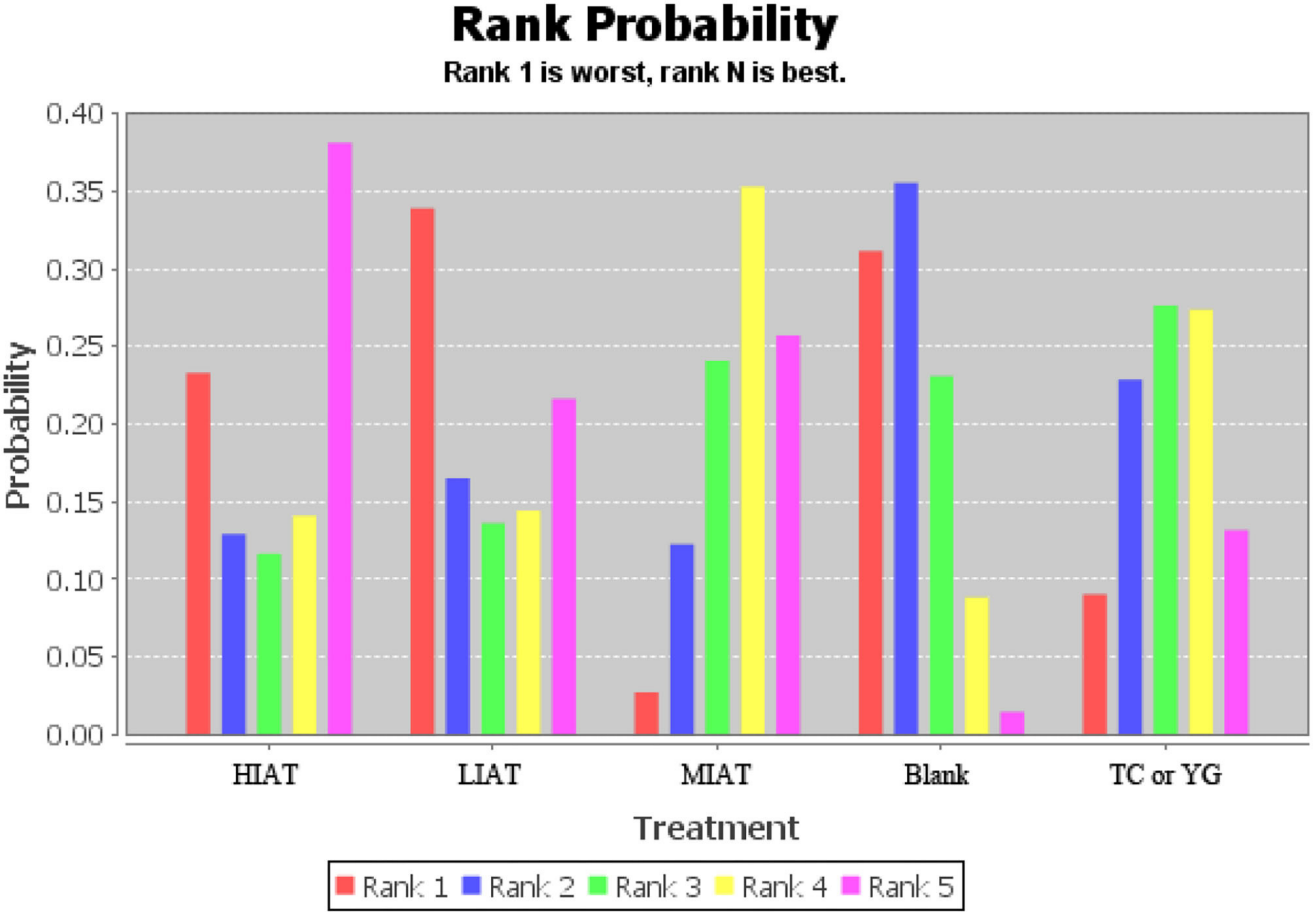
Measurement of malondialdehyde (MDA) and ranking of intervention probability. Blank, Blank control; HIAT, High intensity aerobic training; MIAT, Medium intensity aerobic training; LIAT, Low intensity aerobic training; TC or YG, Tai Chi or Yoga.

The ranking of measurements and probabilities is shown in the bar chart of Figure 4. It should be noted that because the smaller the MDA level, the better the situation. In the ranking probability chart, ranking one is the worst, and ranking N is the best. The results show that for unhealthy people, the means to reduce the level of MDA from the best to the worst are HIAT, MIAT, Tai Chi or Yoga, Blank and LIAT.

The node splitting analysis results would be provided in Table 3, which shows direct evidence, circumstantial evidence, combinatorial evidence, and the estimated quantile of the *P*-value. Excessive *P*-values indicate that no apparent inconsistencies have been found. According to Table 3, the *P*-value is more significant than 0.05, meaning that the consistency model should be used.

**Table 3:** The results of the node splitting analysis.

**Name**	**Direct effect**	**Indirect effect**	**Overall**	***P*-value**
MIAT, TC or YG	−0.03 (−7.86, 8.15)	0.91 (−7.11, 10.15)	0.17 (−2.82, 4.61)	0.77

*MIAT, Medium intensity aerobic training; TC or YG, Tai Chi or Yoga*.

#### Antioxidant Marker

**Figure 5:**
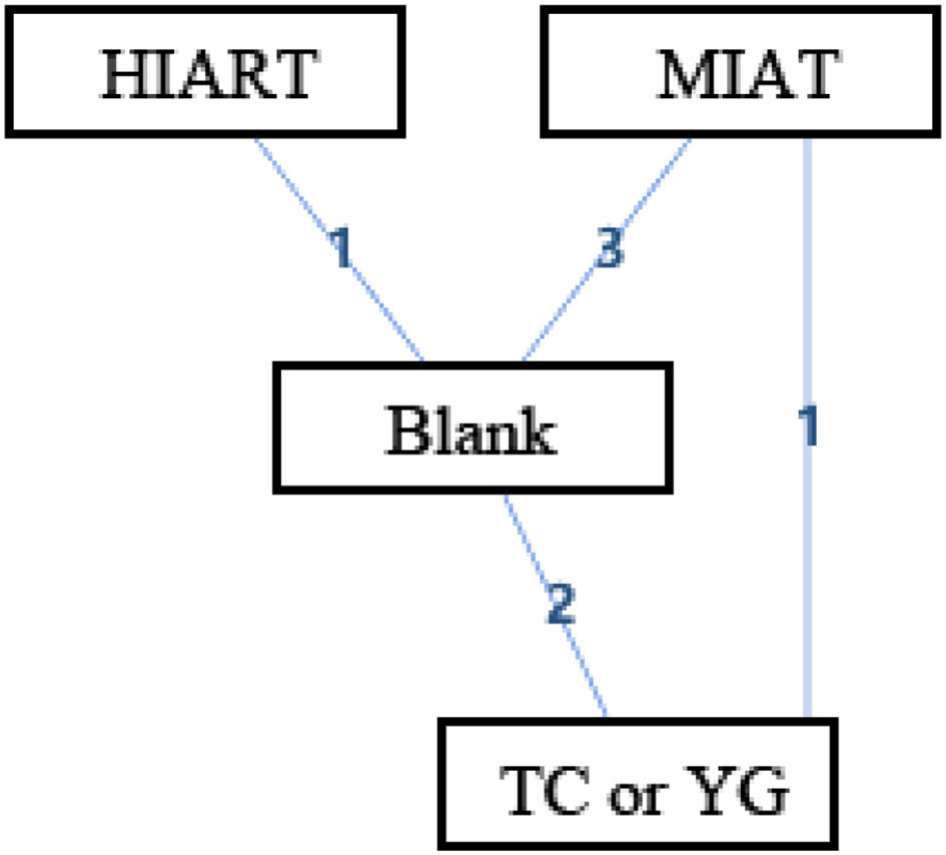
The network structure of intervention of superoxide dismutase (SOD). Blank, Blank control; HIART, High intensity aerobic mixed resistance training; MIAT, Medium intensity aerobic training; TC or YG, Tai Chi or Yoga. The numbers on lines represent the number of studies that make direct comparisons between interventions.

Figure 5 shows the geometry of the acute intervention network of SOD, a biomarker of antioxidant capacity in an unhealthy population. As shown in the figure, there is a mixed intervention comparison (Figure 5). Since the evidence had a closed-loop, the inconsistency of the evidence should be evaluated first.

In the mixed comparison of MIAT, Blank, high intensity aerobic mixed resistance training (HIART) and Tai Chi or Yoga, the random effect standard deviation of the consistency model and its 95% confidence interval was 1.95 (0.93, 3.32), the random effect standard deviation of the inconsistency model and its 95% confidence interval was 1.97 (0.94, 3.32). The inconsistency model's standard inconsistency deviation and 95% confidence interval were 1.56 (0.08, 3.32). In addition, the inconsistency factors of MIAT, Blank, and Tai Chi or Yoga were −0.23 (−3.60, 2.45). The random effect standard deviation of the consistency model and inconsistency model is the same. The inconsistency factor in the intervention cycle is close to 0. It means that there may be consistency differences related to specific nodes, and node split analysis is required.

Table 4 is a sorted table of the network geometry of Figure 5.

**Table 4:** The League Table of the interventions for the superoxide dismutase (SOD).

HIART			
−1.85 (−6.86, 3.09)	MIAT		
0.01 (−4.35, 4.34)	1.85 (−0.56, 4.37)	Blank	
−2.18 (−7.50, 2.96)	−0.35 (−3.67, 3.00)	−2.19 (−5.20, 0.68)	TC or YG

*Blank, Blank control; HIART, High intensity aerobic mixed resistance training; MIAT, Medium intensity aerobic training; TC or YG, Tai Chi or Yoga*.

**Figure 6:**
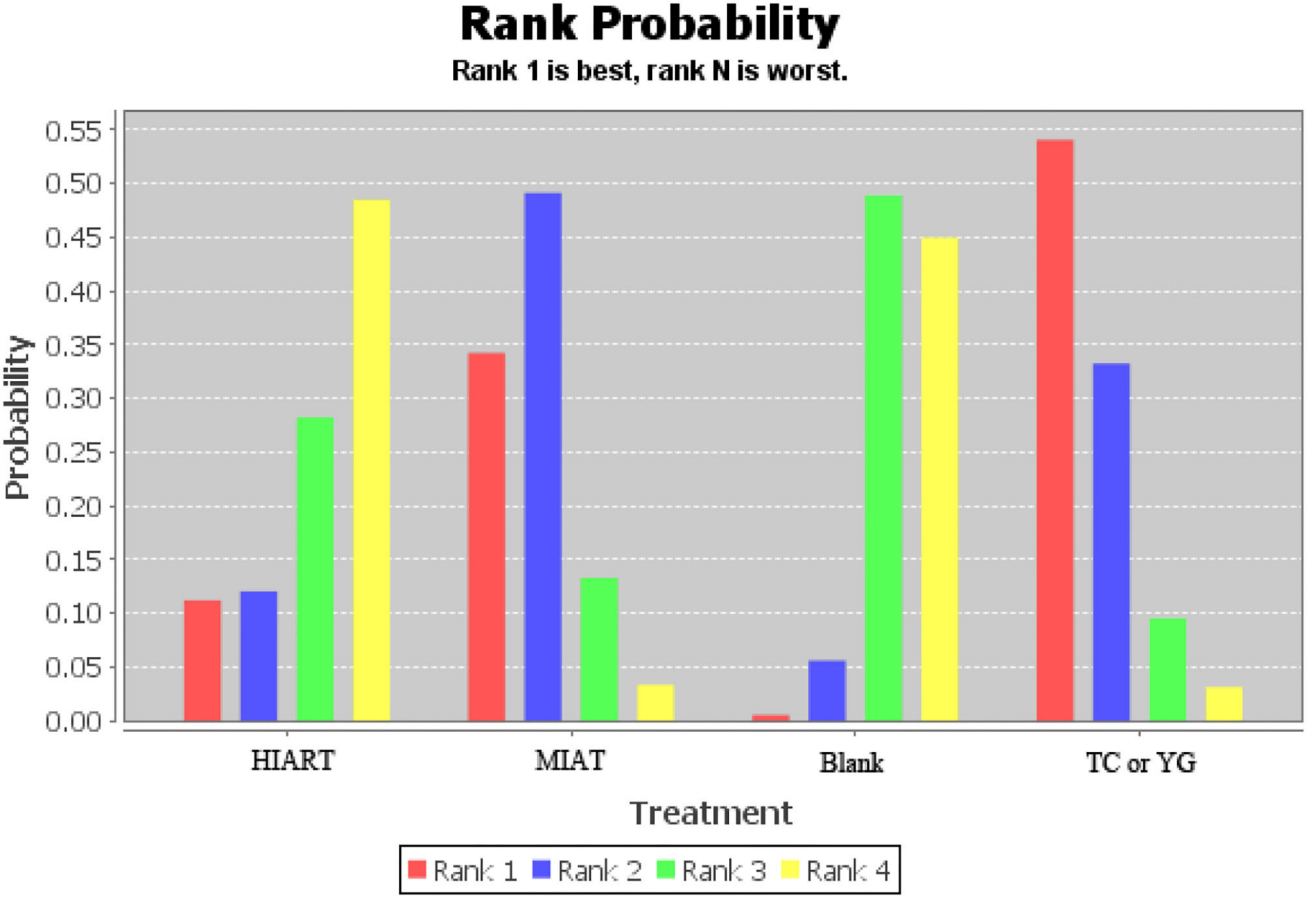
Measurement of superoxide dismutase (SOD) and ranking of intervention probability. Blank, Blank control; HIART, High intensity aerobic mixed resistance training; MIAT, Medium intensity aerobic training; TC or YG, Tai Chi or Yoga.

The ranking of measurements and probabilities is shown in the bar chart of Figure 6. The results show that for unhealthy people, the means to improve the level of SOD from the best to the worst are Tai Chi or Yoga, MIAT, Blank and HIART.

The node splitting analysis results would be provided in Table 5, which shows direct evidence, circumstantial evidence, combinatorial evidence, and the estimated quantile of the *P*-value. Excessive *P*-values indicate that no apparent inconsistencies have been found. According to Table 5, the *P*-value is more significant than 0.05, meaning that the consistency model should be used.

**Table 5:** The results of the node splitting analysis.

**Name**	**Direct effect**	**Indirect effect**	**Overall**	***P*-value**
MIAT, TC or YG	0.34 (−4.31, 4.95)	−0.53 (−5.80, 4.72)	0.35 (−3.00, 3.67)	0.75

*MIAT, Medium intensity aerobic training; TC or YG, Tai Chi or Yoga*.

## Discussion

In this review, the network meta-analysis method was used to make a mixed and indirect comparison of exercise and mixed antioxidants. This study aimed to determine the effects of different types and intensities of exercise on OS and antioxidant capacity in unhealthy populations. By exercise intervention classification, 11 randomized controlled trials conducted a total of 6 different interventions: HIAT, MIAT, LIAT, HIART, Tai Chi or Yoga and Blank. The subjects included patients with type 1 diabetes (Boff et al., 2019), type 2 diabetes (Gordon et al., 2008; Wycherley et al., 2008; Chen et al., 2010; Mallard et al., 2017), chronic kidney disease (Wilund et al., 2010; Gordon et al., 2013), chronic heart failure (Linke et al., 2005), coronary heart disease (Luk et al., 2012), irritable bowel syndrome (Maleki et al., 2018) and severe depression (Schuch et al., 2014).

MDA was reported in 8 studies (Fatouros et al., 2008; Chen et al., 2010; Wilund et al., 2010; Luk et al., 2012; Gordon et al., 2013; Mills et al., 2013; Mallard et al., 2017; Thirupathi et al., 2021), 7 of the studies showed a significant decrease in MDA levels from baseline after exercise training (*p* < 0.05); SOD was reported in 5 studies (Linke et al., 2005; Gordon et al., 2008, 2013; Luk et al., 2012; Maleki et al., 2018), in 3 of the studies, SOD levels increased significantly from baseline after exercise training (*p* < 0.05).

The results show that for patients with chronic diseases with stable health, the appropriate intensity of physical exercise is beneficial. This result was supported because training almost positively affected OS in varying degrees in the included studies. Furthermore, there were few reports of adverse effects of exercise as an intervention. And the subjects in the study, after receiving different intensity and cycle of exercise intervention, blood pressure, cardiopulmonary function, exercise ability, blood lipids, and endothelial function were improved in varying degrees.

It is worth noting that the blank control is not the worst among the intervention rankings of lowering MDA and improving SOD. According to our results, LIAT is the worst in reducing MDA. In the only study in which LIAT was conducted (Chen et al., 2010), LIAT was conducted in the form of calisthenics, and the oxidative stress status of the subjects did not change significantly after a period of intervention, which may be due to the low intensity of the intervention. In improving the effect of SOD, HIART is the worst. In the studies we included, only one study received HIART intervention (Luk et al., 2012). The intervention results showed that there was no significant change in the level of oxidative stress in the subjects, which may be related to excessive exercise intensity or the special pathological conditions of patients with coronary heart disease. In addition, in the study of Gordon et al. (2013), the oxidative stress of the blank control group also observed a significant improvement, and the subjects underwent hemodialysis before blood collection, which may have an impact on the results of the intervention. In the study of Wycherley et al. (2008), the oxidative stress status of the blank control group was also observed to be significantly improved, and the subjects not only received exercise intervention but also carried out diet control, which may lead to the improvement of oxidative stress in the control group. Therefore, although the intervention effect of LIAT or HIART is not as effective as that of the blank control in our results, this may be due to too few studies of LIAT and HIART intervention or other interventions (such as diet control) in the blank control group. More studies may be needed to verify the effectiveness of LIAT and HIART interventions.

It should be noted that although most of the evidence suggests that exercise plays a positive role in OS in unhealthy people, excessively intense exercise may increase OS and reduce the production of antioxidant enzymes in the short term (Pingitore et al., 2015; Thirupathi et al., 2021). This acute stress response has harmful effects on patients with specific diseases (Fatouros et al., 2008). Therefore, it is necessary to recommend appropriate exercise for unhealthy people according to the type and intensity of exercise. In addition, different diseases are heterogeneous. For example, type 2 diabetes will destroy patients' oxidative resistance and increase patients' OS (Jain and McVie, 1994; Obrosova et al., 2002). Therefore, it is necessary to discuss the influence of exercise on OS according to the type of disease.

### Age

OS is associated with some aspects of the aging process and plays an essential role in many age-related diseases (Khansari et al., 2009; Edrey and Salmon, 2014). Regular exercise may improve people's OS, and the effect may vary according to age.

In the study of Boff et al. (2019), 27 young patients with diabetes (mean age: 23.5 years) received two different intensity aerobic training interventions (HIAT and MIAT). Unfortunately, the results showed that OS did not improve (Boff et al., 2019). In contrast, in Maleki et al.'s study, 51 young women (mean age: 34 years old) received 24 weeks of MIAT, OS was significantly improved (p <0.05) (Maleki et al., 2018).

In the study of Linke et al. (2005) and Mallard et al. (2017), 59 middle-aged and older people (mean age: 55.7 years old) had no significant improvement in OS after a period of MIAT or HIAT.

In the study of Chen et al. (2010) and Gordon et al. (2008), a total of 325 middle-aged and older people aged between 40 and 70 years old (mean age 62.2 years old) participated in the study. The subjects showed significant improvement in OS after a period of Tai Chi, Yoga, and MIAT (*p* < 0.05).

In the study of Luk et al. (2012), 64 older people took part in the experiment. After 8 weeks of HIART, there was no significant improvement in OS.

In addition, 4 studies were conducted on unhealthy people of all ages (subjects aged between 18 and 70, with an average age of 44.38) (Wycherley et al., 2008; Wilund et al., 2010; Gordon et al., 2013; Schuch et al., 2014). In these studies, a total of 140 subjects participated in the study. After a period of MIAT or Yoga, OS was significantly improved (*p* < 0.05).

Among the 11 studies included, no difference was found in exercise on OS in unhealthy people of different ages. However, none of these studies are directly related to age-related studies, so the subject cannot be analyzed more accurately, and more studies on age can be carried out in the future.

### Exercise Intensity

Long-term exercise training is known to reduce OS (De Sousa et al., 2017) and improve the antioxidant capacity of cells (Polidori et al., 2000; Fisher-Wellman and Bloomer, 2009). However, different levels of exercise produce other effects, especially for unhealthy people with chronic diseases, the balance between OS and antioxidants remains controversial (Parker et al., 2014; Vezzoli et al., 2014).

#### High-Intensity Training

The high-intensity exercise intervention was used in 3 of the 11 studies (Luk et al., 2012; Mallard et al., 2017; Boff et al., 2019). The three studies were consistent in terms of the effects of high-intensity exercise training on OS and antioxidant parameters in unhealthy individuals. In the study of Boff et al., 9 subjects with type 1 diabetes showed no statistically significant change in their OS level of MDA after 8 weeks of HIAT compared to 8 weeks before (*p* > 0.05). In the study of Luk et al., 32 subjects with coronary heart disease underwent 8 weeks of HIART, and there was no statistically significant change in SOD and 8-ISO of antioxidant capacity indicators (*p* > 0.05). In the study of Mallard et al., 20 subjects with type 2 diabetes who underwent 52 weeks of HIAT, there were no statistically significant changes in OS markers PC and antioxidant capacity markers TEAC, GPX, and F2-iso (*p* > 0.05).

Although we have obtained evidence that high-intensity physical exercise over some time may not be effective in lowering OS and improving their antioxidant capacity in unhealthy people. It's important to note that these groups can benefit in other ways from intense physical activity. For example, in Boffw et al.'s study, improvements in endothelial function were observed during 8 weeks of training. In Luk et al.'s study, exercise training improved endothelial function, increased HDL-C levels, and reduced resting heart rate and diastolic blood pressure in patients with CHD; however, discussion of these results is beyond the scope of this article. In conclusion, there is no evidence that high-intensity exercise training can improve the REDOX status of unhealthy people. However, only three studies have investigated the effects of high-intensity exercise training on OS parameters, and the results are few. More studies are needed to draw practical conclusions.

#### Moderate Intensity Training

Eight of the 11 studies included in this review examined the effects of MIAT on OS in unhealthy people (Linke et al., 2005; Gordon et al., 2008; Wycherley et al., 2008; Wilund et al., 2010; Schuch et al., 2014; Mallard et al., 2017; Maleki et al., 2018; Boff et al., 2019). Results from 5 of the studies showed significant improvements in REDOX status after intervention (*p* < 0.05) (Gordon et al., 2008; Wycherley et al., 2008; Wilund et al., 2010; Schuch et al., 2014; Maleki et al., 2018). Three studies reported unchanged REDOX status after intervention (*p* > 0.05) (Linke et al., 2005; Mallard et al., 2017; Boff et al., 2019).

Although the results were inconsistent, most studies showed an improvement in subjects' OS status after moderate exercise intervention over time, and other studies showed no deterioration in their OS status. Therefore, in practical application, MIAT can be used in unhealthy people to reduce OS and delay the onset of OS-related diseases. In addition, in this review, all the intervention methods of moderate-intensity exercise were aerobic training, so it is impossible to determine the influence of other moderate-intensity exercise types on the OS status of unhealthy people, and more studies are needed to conclude.

#### Low-Intensity Training

Three studies (5 interventions) reported the effects of low-intensity exercise interventions on OS in unhealthy people, and 3 of these interventions produced significant improvements in subjects' OS status (*p* < 0.05) (Gordon et al., 2008, 2013; Chen et al., 2010).

In the study of Chen et al., 12 weeks of Tai Chi significantly reduced the MDA level of subjects. In comparison, 12 weeks of aerobic training did not observe statistically significant changes in the subjects' OS indicators (Chen et al., 2010). In Gordon et al.'s study, 16 weeks of Yoga training significantly improved subjects' OS (Gordon et al., 2013). In the study of Gordon et al., 12-week Yoga did not significantly improve the subjects' OS. Still, when the intervention time reached 24 weeks, Yoga significantly reduced the MDA level and increased the SOD level of the issues (Gordon et al., 2008). Most studies have shown that low-intensity exercise intervention can improve the OS of unhealthy people. When the duration of the intervention is longer than 12 weeks, the OS of the subjects has different degrees of improvement. It may be speculated that long-term low-intensity exercise intervention can improve OS in unhealthy people, and more long-term experiments are needed to verify this in the future.

### Types of Training

It is well-known that aerobic training and resistance training use different energy systems. When performing aerobic training, time and intensity of training are usually considered, capacity and weight are typically considered when completing resistance training (De Sousa et al., 2017). Therefore, different types of exercise may produce different results.

#### Aerobic Training

Of the included studies, 8 (11 interventions) used aerobic training interventions, and 6 of these interventions significantly improved subjects' OS (Gordon et al., 2008; Wycherley et al., 2008; Wilund et al., 2010; Schuch et al., 2014; Maleki et al., 2018); Subjects' OS improved after 3 interventions. Still, there were no statistically significant change (Linke et al., 2005; Gordon et al., 2008; Mallard et al., 2017); only two interventions showed the opposite result, with MDA levels rising after aerobic training, but not significantly (Boff et al., 2019).

In summary, there is strong evidence to support the positive effect of aerobic training on OS in unhealthy people. The included studies used different aerobic training equipment (e.g., treadmills, power bikes), intensity, and capacity. There has been very little research on the effect of aerobic training on OS in unhealthy people, and more research in this area is needed in the future.

#### Aerobic Mixed Resistance Training

Only 1 of the included studies used aerobic mixed resistance training, and in this study, there was no statistically significant difference in subjects' OS status after training from baseline and control (Luk et al., 2012). However, there are too few studies to draw definitive results, and more research is needed in the future to draw valid conclusions.

#### Tai Chi or Yoga

Tai Chi or Yoga was used in 3 of the included studies (Gordon et al., 2008, 2013; Chen et al., 2010). Two studies reported that Tai Chi and Yoga significantly improved OS in unhealthy people (Chen et al., 2010; Gordon et al., 2013). Subjects in 1 study showed no significant improvement in OS after 12 weeks of Yoga intervention and significant improvement in OS after 24 weeks of Yoga intervention (Gordon et al., 2008).

These results suggest that both Tai Chi and Yoga can improve OS in unhealthy people. In addition, other studies have shown that Tai Chi and Yoga can help reduce psychological stress (Zou et al., 2018), reduce anxiety (Field et al., 2010), and improve the balance ability of the elderly (Hakim et al., 2010). Therefore, it seems that Tai Chi or Yoga could be used in practice to improve OS in unhealthy people, but the current research is still scarce, and more research is needed to provide evidence.

### Type of Disease

#### Type 1 Diabetes and Type 2 Diabetes

Only 1 study investigated the effect of exercise on OS in patients with type 1 diabetes (Boff et al., 2019). In this study, 3 groups of patients with type 1 diabetes received 8 weeks of HIAT and MIAT, respectively. There was no significant difference in OS from baseline in either group or control group. OS is known to cause endothelial dysfunction in type 1 diabetes (Bertoluci et al., 2015). However, in this study, exercise training did not improve OS in subjects. At present, there are few studies on the influence of exercise training on OS in patients with type 1 diabetes. Furthermore, the issues in this study are all young patients with diabetes complications, so it is impossible to infer the whole population of patients with diabetes. Therefore, it is insufficient to confirm whether exercise training can cause OS changes in patients with type 1 diabetes.

Four studies examined the effect of exercise training on OS in patients with type 2 diabetes (Gordon et al., 2008; Wycherley et al., 2008; Chen et al., 2010; Mallard et al., 2017). Chen et al. (2010) studied 94 patients who underwent 8 weeks of two different types of physical exercise (Tai Chi and LIAT), in which the subjects who received the Tai Chi exercise intervention showed a significant decrease in MDA levels (*P* < 0.05), indicating a possible improvement in OS. Gordon et al. (2008) conducted 24 weeks of different types of physical exercise (MIAT and Yoga) on 231 patients and reported that there was no significant change in the patients' OS at 12 weeks (*P* > 0.05), MDA level decreased (*P* < 0.05) and SOD level increased (*P* < 0.05) at 24 weeks. It indicated that the patient's OS decreased and the antioxidant capacity increased. Hakim et al. (2010) studied 29 women, and MDA levels were significantly reduced after 12 weeks of training (*P* < 0.05), indicating that patients' OS may have been improved to some extent.

On the other hand, in the study of Mallard et al. (2017), 36 patients with type 2 diabetes who underwent 52 weeks of HIAT or MIAT showed no significant changes in OS and antioxidant indices (*p* > 0.05). Notably, when grouped by gender, the PC level of subjects receiving MIAT decreased significantly (*P* < 0.05), suggesting that exercise training may positively affect type 2 diabetes patients, and the effect varies among patients of different genders. However, there are very few studies on the effect of exercise training on OS in patients with type 2 diabetes of different genders, and more studies are needed to draw valid conclusions.

#### Chronic Heart Failure and Coronary Heart Disease

One study (Linke et al., 2005) investigated the effects of MIAT on patients with chronic heart failure, and the other (Luk et al., 2012) investigated the impact of HIART on patients with coronary heart disease. No significant changes in subjects' OS and antioxidant capacity were observed (*p* > 0.05).

In the study by Linke et al. (2005), a modest increase in both GPX and Catalase levels was observed after 24 weeks of training, but not statistically significant changes, which may indicate that 24 weeks of MIAT was not sufficient to produce a significant improvement in OS in patients with chronic heart failure. In Luk et al.'s study (Luk et al., 2012), 64 patients with coronary heart disease who received 8 weeks of HIAT showed significant improvement in their health status and exercise ability but no change in antioxidant capacity (*P* > 0.05).

These results do not prove that exercise training improves OS in patients with chronic heart failure and coronary heart disease, but no adverse events were observed, and patients' health was improved. Cardiovascular diseases are usually multifactorial, and the patients are heterogeneous (Lopez-Suarez et al., 2014). Currently, there are still too few studies, and more evidence is needed to draw practical conclusions.

#### Chronic Kidney Disease

Two studies have investigated the effect of exercise training on OS in patients with chronic kidney disease (Obrosova et al., 2002; Vezzoli et al., 2014). Significant improvement in OS was observed after 16 weeks of Yoga and MIAT (*p* < 0.05).

Evidence suggests that exercise training may improve OS in patients with chronic kidney disease and provide other benefits. These data provide evidence that exercise training improves OS in patients with chronic kidney disease, but we need to evaluate these results with more large-scale and longer-duration interventions.

#### Irritable Bowel Syndrome

One study investigated the effect of MIAT on OS in patients with irritable bowel syndrome (Maleki et al., 2018), and 51 patients participated in the experiment. Results showed that OS was significantly improved after 12 weeks of exercise intervention (*P* < 0.05), and the change was even greater after 24 weeks of intervention and showed a significant difference compared with 12 weeks (*p* < 0.05).

The results suggest that more than 12 weeks of MIAT is sufficient to inhibit oxidant production and systematically adapt the antioxidant system in patients with irritable bowel syndrome. These improvements may be related to improved bowel function in patients with irritable bowel syndrome (Maleki et al., 2018). In summary, the obtained data support the idea that exercise training can improve OS in patients with irritable bowel syndrome.

#### Major Depression

One study investigated the effect of exercise training on OS in patients with major depressive disorder, involving 26 patients (Schuch et al., 2014). After 3 weeks of MIAT, the MDA level was significantly decreased (*P* < 0.05).

Since the study had an additional design, the decrease in MDA levels may be related to the synergistic effects of exercise training and antidepressant use (Schuch et al., 2014). Although there was only one study looking at the impact of exercise on OS in patients with major depression, the 3-week intervention did reduce MDA levels in patients, suggesting that exercise training could be used to improve OS in this population.

### Limitations

Due to the high degree of clinical heterogeneity and the scarcity of data, our analysis cannot be extrapolated to the whole population with pathology. However, a similar trend was observed in OS in patients with heart failure (Meirelles et al., 2014; Sties et al., 2018), stroke (Gambassi et al., 2019), male infertility (Maleki and Tartibian, 2017), and COPD (Mercken et al., 2009) who received exercise intervention. So far, it has been proved that physical exercise can improve OS and bring health benefits (Powers et al., 2020; Thirupathi et al., 2021). However, according to our systematic review and meta-analysis results, there was no significant improvement in OS after HIART (Luk et al., 2012; Mallard et al., 2017; Boff et al., 2019).

Limitations of the study: (1) OS is a complex process, and our reticular meta-analysis is only a reductionist method for this phenomenon; (2) the number of articles included in the reticular meta-analysis is limited, and publication bias cannot be evaluated; (3) insufficient sample size may lead to overestimation of the effectiveness of the intervention; (4) All patients with chronic diseases may use pro-oxidants and antioxidants. However, there are too few studies on the synergistic effect of exercise and related drugs at present. In addition, some exercise interventions are conducted only once. Hence, it is impossible to evaluate the effect of long-term exercise intervention combined with related drugs; (5) Studies have found that there are gender differences in the influence of exercise on OS in unhealthy people. Still, there is almost no gender division in the included literature, so it is impossible to analyze the image of the effect of exercise intervention by gender; (6) The outcome indicators of the included literature were different, and the methods for detecting relevant indicators were also different, so it was impossible to conduct a reticulated meta-analysis of all hands; (7) In recent years, there are still very few studies on the effects of exercise on the OS of unhealthy people, and there is no study on anti-resistance training, so it is impossible to conduct a more comprehensive analysis on various types of exercise with different intensities.

## Conclusion

In this network meta-analysis, MDA and SOD were the main indicators of OS and antioxidant capacity. The OS and antioxidant levels of subjects after intervention were taken as the main results. The effects of different intensities and types of physical exercise on OS in unhealthy people were compared indirectly. The verified consistency model is applied to network meta-analysis. To sum up, HIAT and Tai Chi or Yoga may be the best choice to reduce OS in unhealthy people. Moreover, exercise training to improve OS seems to be different among different types of patients. When recommending exercise therapy for patients, the type and intensity of exercise should be other according to the type and severity of the disease. The use of various drugs in conjunction with exercise intervention may lead to misjudgment. The results show great potential for conservative treatment of OS in unhealthy people through exercise training. However, there are still few studies on the effects of an exercise intervention on OS in unhealthy people, and more high-quality studies are needed to evaluate the effects of different intensity and types of exercise training in long-term intervention treatment.

## Data Availability Statement

The original contributions generated for the study are included in the article/supplementary material, further inquiries can be directed to the corresponding author/s.

## Author Contributions

ZL, YX, and YS conceived the presented idea, developed the framework, and wrote the manuscript. IB and YG provided critical feedback and contributed to the final version. All authors were involved in the final direction of the paper and contributed to the final version of the manuscript and have read and agreed to the published version of the manuscript.

## Conflict of Interest

The authors declare that the research was conducted in the absence of any commercial or financial relationships that could be construed as a potential conflict of interest.

## Publisher's Note

All claims expressed in this article are solely those of the authors and do not necessarily represent those of their affiliated organizations, or those of the publisher, the editors and the reviewers. Any product that may be evaluated in this article, or claim that may be made by its manufacturer, is not guaranteed or endorsed by the publisher.
